# Cryo-EM Structure of the FtsH Periplasmic Domain Reveals Functional Dynamics

**DOI:** 10.1021/acschembio.5c01025

**Published:** 2026-04-07

**Authors:** Günce Göc, Sathish K. N. Yadav, George Orriss, Ufuk Borucu, Imre Berger, Christiane Schaffitzel, Burak V. Kabasakal

**Affiliations:** † Turkish Accelerator and Radiation Laboratory, Ankara 06830, Türkiye; ‡ Department of Biology, Kocaeli University, Kocaeli 41001, Türkiye; § School of Biochemistry, 1980 University of Bristol, Bristol BS8 1TD, U.K.; ∥ School of Chemistry and Max Planck Bristol Centre for Minimal Biology, University of Bristol, Bristol BS8 1TH, U.K.; ⊥ Department of Biological Sciences, Middle East Technical University, Ankara 06800, Türkiye

## Abstract

FtsH, an AAA + metalloprotease that is essential in bacteria and eukaryotic organelles, maintains cellular homeostasis by degrading misfolded and membrane-associated proteins. Here, we report cryo-EM structures of the *Escherichia coli* FtsH periplasmic domain (FtsH-PD) revealing insights into its intrinsic conformational flexibility. Our analysis resolved two distinct states: a 4.9 Å structure exhibiting the conserved α + β fold and a 7.3 Å map representing distinct rotated-helix conformation characterized by 20° clockwise rotation of two alpha helices. These findings support a model where conformational changes are present not only in the FtsH cytosolic domain but also in the periplasmic domain. This flexibility potentially facilitates substrate translocation through a combination of mechanisms involving both the FtsH-PD and the HflKC complexed with FtsH, along with lipid–scramblase activity, to assist in membrane protein extraction. This study offers new perspectives on how conformational changes in the periplasmic domain contribute to FtsH substrate degradation mechanisms.

## Graphical abstract

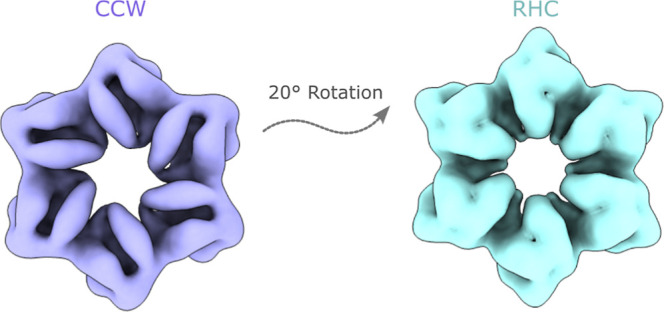

## Introduction

The AAA+ (ATPases associated with diverse cellular activities) protein family consists of universally important hexameric or heptameric oligomers involved in various cellular processes, such as DNA replication, membrane fusion, and signal transduction.^1^ Within this broad family, AAA+ proteases form a specialized subgroup that plays a crucial role in maintaining cellular homeostasis, specifically through the targeted degradation of misfolded or unfolded proteins. The degradation of protein substrates by AAA+ proteases occurs in two stages regarding the two domains of AAA+ Proteases: the ATPase domain provides the mechanical energy derived from the conversion of chemical energy using ATP, which is then employed to unfold the three-dimensional structure of the target protein in the proteolytic domain. Subsequently, the degraded target protein is decomposed into polypeptide chains and amino acids.^2^

The degradation process of the target proteins is a crucial mechanism for the maintenance of protein homeostasis, which is why it is termed protein quality control. As the most significant member of the protein quality control mechanism with considerable substrate variability, FtsH (Filamentation Temperature Sensitive Protein H) plays a pivotal role in maintaining cellular homeostasis as a AAA+ metalloprotease. The uniqueness of FtsH comes from being an integral membrane protein and its ability to degrade both membrane-bound and soluble proteins. Consequently, FtsH is capable of degrading a wide range of substrates, thereby facilitating the involvement of various metabolic processes.^3–5^

As a widely conserved hexameric AAA+ metalloprotease, each *Escherichia coli* FtsH subunit has a cytosolic domain (FtsH-CD) with ATPase and proteolytic activity and a periplasmic domain (FtsH-PD) in a single polypeptide chain^3,6^ (Figure 1). The N-terminal part comprising two transmembrane helices is followed by the AAA+ ATPase domain, which contains Walker A (amino acid sequence: GPPGTGKT) and Walker B motifs (amino acid sequence: VAGCDE). These motifs coordinate binding and hydrolysis of ATP, with the participation of bound Zn^2+^ and H_2_O. To provide mechanical energy for substrate translocation, hydrolysis of ATP is necessary. ATP hydrolysis results in a “pulling” which facilitates conformational changes in the cytoplasmic domain of FtsH, causing the substrate to move forward through the central cavity of the proteolytic domain, leading to degradation of the bound substrate into small oligopeptides.^7,8^

**Figure 1:**
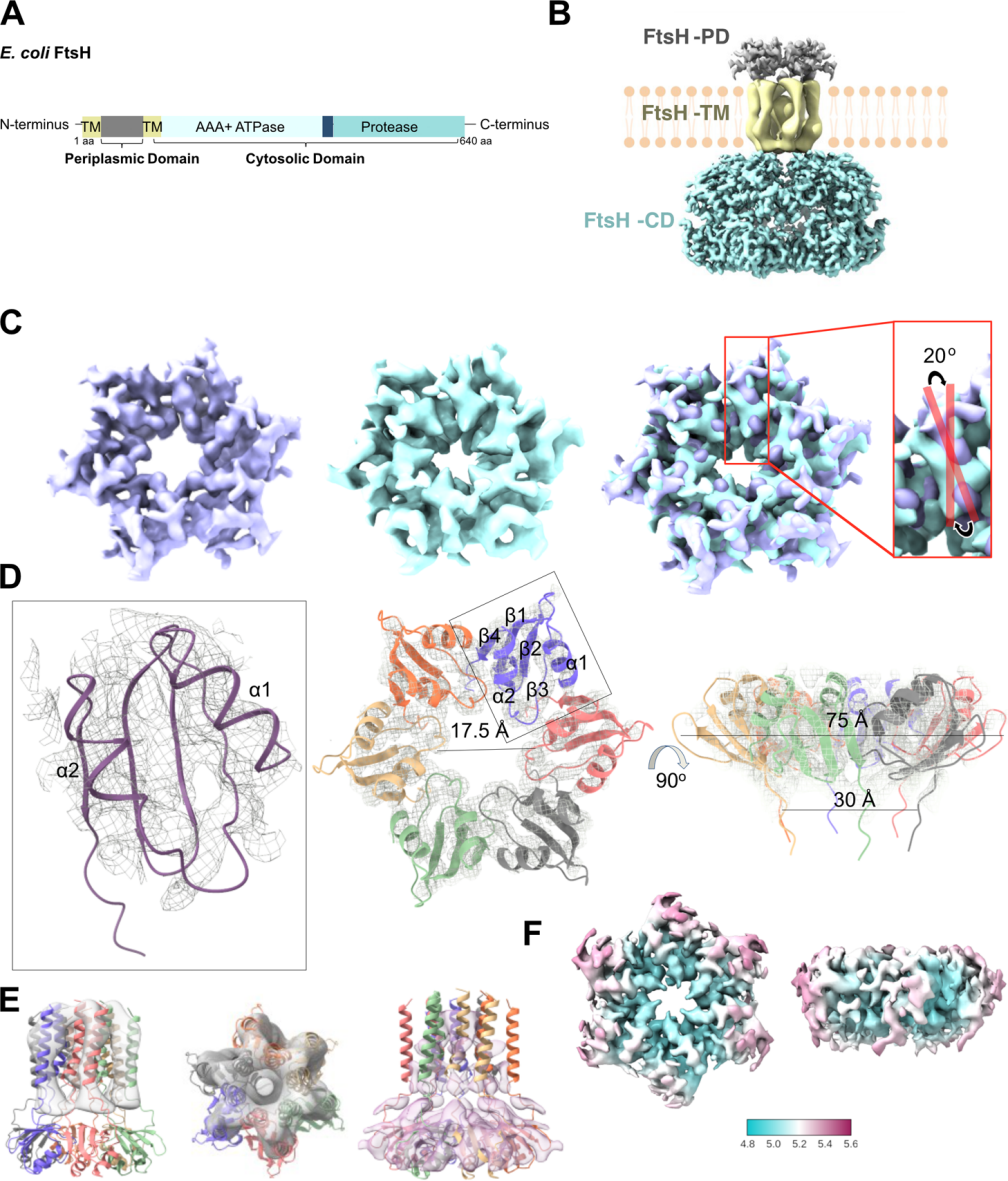
Structural Analysis of *E. coli*FtsH. (A) Domain architecture and structure of*E. coli*FtsH domains. Schematic representation of the domain architecture of *E. coli* FtsH. The protein comprises an N-terminal periplasmic domain (gray), two transmembrane helices (yellow), a cytosolic AAA+ ATPase domain (light blue), and a protease domain (turquoise). (B) Structural model of the FtsH complex integrated into the membrane. The periplasmic domain (FtsH-PD, gray), transmembrane helices (FtsH-TM, yellow), and cytosolic domains (FtsH-CD, turquoise) are depicted, based on the maps from this study (PD and TM) and the EMD-32521 ^22^ (CD). (C) Counterclockwise (CCW) (purple) and rotated-helix conformation (RHC) (turquoise) maps of FtsH-PD. Superposition of maps shows that FtsH-PD undergoes a conformational change by a 20° clockwise rotation, highlighted by sticks on the α2 helices. (D) Hexameric model of the FtsH-PD. Atomic model fitted into the cryo-EM map (gray mesh). One subunit is highlighted (box). Alpha helices undergoing a conformational change are labeled as α1 and α2. (E) FtsH-TM maps. AlphaFold2 model of the hexameric FtsH-PD-TM fitted into FtsH-TM maps reconstructed from subtracted particles (gray), with a loose mask (salmon). (F) Local resolution map of FtsH-PD, indicating a resolution ranging from 4.8 to 5.6 Å.

Structures of ADP-bound FtsH from *Thermotoga maritima* ^5^ and *Aquifex aeolicus*,^7^ and an ATP-bound yeast homologue YME1 structure have been determined.^9^ The crystal structures of the FtsH-CD indicated that the ATPase domain can exhibit C3 ^10^, C2 ^11^, and C6 ^12^ symmetry, adopting different conformations depending on the identity of the bound nucleotide (ATP, ADP), or lack thereof (apo structure). This nature of these conformational changes has led to the current consensus that the “pulling” mechanism is most probable.^12^ In addition to crystal structures, a cryo-EM structure of the FtsH-CD homologue, YME1 (pdb 6az0), exhibited a “staircase” symmetry of the ATPase domain,^9^ meaning the subunits are arranged in a spiral, step-like configuration that facilitates substrate translocation. This structure indicates an opening of 1.4 nm, which would be sufficient for the passage of small peptides. Comparison of mitochondrial inner membrane protease YME1-CD with the apo-state structure of *T. maritima* FtsH-CD (pdb 2ce7) shows that the ATPase domain is capable of rotating 28° to facilitate the access of peptides to the central pore, further supporting the “pulling” hypothesis.^7,9^ While a recent cryo-EM study reported the structure of the entire FtsH–HflKC complex, it captured the FtsH periplasmic domain in a single state. The effect of FtsH cytosolic domain conformational changes on the periplasmic domain of FtsH, or possible intrinsic conformational dynamics in the periplasmic domain, remains unclear.

Here, we report the structural characterization of *E. coli* FtsH-PD, which we obtained from the purification of FtsH–HflKC complexes using electron cryo-microscopy (cryo-EM). The FtsH-PD exhibits C6 symmetry. Our cryo-EM analysis revealed two different orientations of FtsH-PD, named counterclockwise (CCW, EMD-66269) and a novel orientation named as rotated-helix conformation (RHC, EMD-66013). These conformations were resolved to resolutions of 4.9 Å and 7.3 Å, respectively. Although 7.3 Å is not high resolution, it provides a secondary structure level detail fundamentally sufficient to unambiguously resolve a rigid-body 20° clockwise rotation of two helices relative to the FtsH-PD-CCW map. The two conformations suggest a molecular mechanism how the periplasmic domain of FtsH may be involved in the proteolysis of membrane-bound substrates.

## Materials and Methods

### Protein Expression and Purification Conditions

Protein expression was carried out by using one single plasmid-carrying gene-encoding FtsH-3 × StrepTag and HflKC-10 × His, each under the control of inducible promoters; arabinose and T7, respectively, which was generated using the ACEMBL system.^13^ *E. coli*C43 (DE3) cells expressing the C-terminal streptactin-tagged FtsH-HflK-HflC were grown overnight at 37 °C with shaking with 100 μg/mL Ampicillin and 50 μg/mL Spectinomycin in Luria–Bertani broth (LB) media. The expression media (2xYT) were inoculated at 1:50 dilution, incubated at 37 °C, shaking until OD at 600 nm reaches 0.6–0.8. Protein expression was inducted with 1 mM Isopropyl-β-*d*-thiogalactopyranoside (IPTG) and 0.2% (w/v) arabinose at 37 °C for 3 h. 0.2 mM ATP was added to the media at the time of induction. At the end of 3 h, cells were harvested by centrifugation at 4500 ×*g* for 25 min. The separated cells were resuspended in Buffer A (20 mM HEPES, pH 8.0, 150 mM KCl, 5 mM beta-mercaptoethanol, 20% (v/v) glycerol). Protease inhibitor tablets were added to resuspended cells, and cells were lysed using a cell disrupter at 25 kpsi by passing twice. Five units/ml of benzonase and RNase were added to lysed cells and spun at 20,000 ×*g* for 40 min at 4 °C to discard pellets. Membranes were isolated by centrifuging the supernatant at 100,000 ×*g* using a Type 45 Ti rotor (Beckman Coulter) for 160 min at 4 °C. Membranes were resuspended in Buffer A using a glass Dounce homogenizer and solubilized in 2% (w/v) *n*-dodecyl-β-*d*-maltoside (DDM) (Sigma-Aldrich) by gently shaking for 2 h at 4 °C. Supernatant containing the membrane proteins was obtained by centrifugation at 100,000*x*g using a Type 45 Ti rotor for 160 min at 4 °C and loaded onto streptactin resin equilibrated with Buffer A’ (20 mM HEPES, pH 8.0, 150 mM KCl, 1 mM beta-mercaptoethanol, 0.01% (w/v) DDM). Proteins were eluted with 4 mM desthiobiotin and concentrated to ∼200 μL using 100 kDa MWCO concentrators.

### ATPase Activity Assay of FtsH and FtsH–HflKC

ATPase activity of membrane protein FtsH was analyzed using the EnzCheck Phosphate Assay Kit (Thermo Fisher Scientific). Measurements were conducted according to the manufacturer’s instructions. A standard calibration curve for P_i_ quantification was generated using a series of reaction mixtures with increasing concentrations of potassium dihydrogen phosphate (KH_2_PO_4_) as the phosphate standard, ranging from 0 to 100 μM final concentration. All reactions were mixed thoroughly and incubated at 22 °C for 30 min. Absorbance was then measured at 360 nm using a UV-3600i Plus UV–vis–NIR spectrophotometer (Shimadzu). The background absorbance from the phosphate-free control was subtracted from all measurements. A standard curve was generated by plotting the known phosphate concentrations (*X*-axis) against the corresponding absorbance values (*Y*-axis), and a linear regression analysis was performed to determine the equation of the trendline, which was subsequently used to quantify Pi generated in experimental samples.

In all ATPase assay conditions, the FtsH and FtsH–HflKC used at a final concentration of 3 μM, along with a substrate solution containing 5 mM ATP, were added to the reactions. Following substrate addition, absorbance at 360 nm was measured at time zero and subsequently at 15 min intervals for a total duration of 60 min. Time-dependent increases in absorbance were recorded and converted to phosphate concentrations using the previously generated standard curve, allowing for quantification of the ATPase activity.

### Protease Activity Assay of FtsH and FtsH–HflKC

The protease activity of FtsH and FtsH–HflKC was measured using resorufin-labeled 0.4% casein (Sigma-Aldrich) as a substrate, and the measurements were conducted in alignment with the manufacturer’s instructions. Reaction was initiated using 0.5 μM FtsH or FtsH–HflKC and aliquots were taken at definite time points of 15, 30, 45, 60, and 120 min. Then, reactions were terminated using 5% TCA, followed by 10 min incubation at 37 °C. Samples were then centrifuged at 12,000 ×*g* for 5 min. 0.5 M Tris–HCl (pH 8.8) was added prior to measuring the absorbance at 574 nm.

### Reconstitution in Amphipol

300 μg of protein at 1 mg mL^–1^ was solubilized in DDM and buffer exchanged into Amphipol (A8-35, Anatrace) at a protein/amphipol mass ratio of 1:7. The protein/amphipol mixture was incubated on a rotator for 2 h at 4 °C. To remove the excess free detergents, 40*X* mass excess of SM2 BioBeads (BioRad) was previously washed with methanol and water, dried, added to the protein/amphipol mixture, and incubated overnight at 4 °C on a rotator. The protein/amphipol/BioBead mixture was centrifuged in a microcentrifuge at 15,000*xg* for 15 min. The supernatant containing the detergent-free and amphipol-reconstituted membrane protein was carefully taken and concentrated in a 0.5 mL concentrator with a 30 kDa MWCO in Buffer C (20 mM HEPES, pH 8.0, 150 mM KCl) to a final volume of 50 μL. The concentrated protein was then injected to a 3.2/300GL Superose 6 column running at 0.05 mL/min of Buffer C, with 0.05 mL fractions collected in a 96-well plate on a micro AKTA.

### Negative Stain EM and Cryo-EM Sample Preparation

Five μL portion of 0.04 mg mL^–1^ protein was applied onto a freshly glow discharged (1 min at 30 mA) carbon film-coated grid (ECF300-Cu; Electron Microscopy Sciences), incubated for 1 min, and manually blotted. Five μL of 3% uranyl acetate was applied onto the same grid and incubated for 1 min before the solution was blotted off. The grid was loaded into a FEI Tecnai12 120 kV BioTwin Spirit TEM. Images were acquired at a nominal magnification of 49 000x with a FEI Ceta camera.

For the Cryo-EM sample, 3 μL of 0.35 mg mL^–1^ protein was applied onto a freshly glow discharged (1 min at 30 mA) Quantifoil R1.2/1.3 carbon grid (Agar Scientific), blotted using a Vitrobot MarkIV (Thermo Fisher Scientific) at 80% humidity and 16 °C for 1.6 s, and plunge frozen.

### Data Collection and Processing of FtsH-PD and FtsH-TM

A data set with 6331 movies was collected with EPU on a Talos Arctica at GW4 Facility, Bristol, UK, with an acceleration voltage of 200 kV and magnification of 130,000*x*, with an effective pixel size of 0.525 Å (binned by 2) in super-resolution mode on a Gatan K2 Summit direct electron detector and Gatan Quantum GIF energy filter operated in zero-loss mode with a slit width of 20 eV. Movies were collected as 48 frames with a defocus range from −1 to −2 μm and a total exposure of 61.3 e-/Å^2^. Cryo-EM data was processed using the Relion 3.1, 4.0, and 5.0 software packages.^14,15^ First, the motion correction of micrographs was performed with MotionCorr2,^16^ and contrast transfer function (CTF) estimation was done using ctffind4.1.^17^ Micrographs with resolutions better than 4.2 Å were selected for particle selection (5469 out of 6331 micrographs). After two rounds of 2D classification, 929,166 particles were used for subsequent periplasmic and transmembrane region reconstructions. First, particles corresponding to either periplasmic or transmembrane regions, 429,744 and 455,307 particles, respectively, were subtracted (Supporting Information Figure S1, Supporting Information, Table S1).

After 3D classification of the periplasmic region-subtracted particles, and in parallel 3D-focused classification rounds using the original extracted particles, three maps with different orientations of periplasmic FtsH were refined; counterclockwise (CCW) orientation with 9590 particles, clockwise (CW) orientation with 20,776 particles, and rotated-helix confirmation (RHC) with 8990 particles. After ctf and aberration refinement, Bayesian polishing, and postprocessing, 5.4 Å map of CCW, 5.2 Å map of CW orientations, and 7.3 Å map of RHC FtsH-PD were acquired. As the CW map consisted of left-handed helices, particles of CCW and CW orientations were combined and re-refined. The final postprocessed right-handed map (CCW) reached up to 4.9 Å based on the Fourier Shell Correlation (FSC) 0.143 cutoff.^18^ Local resolution of the final map was determined in Relion 5.0 and visualized with ChimeraX^19^ (Supporting Information Figure S3). DeepEMhancer was used to generate sharpened maps for visualizations and model building.^20^

For the transmembrane (TM) region, after 3D classification without applying symmetry with subtracted particles, 349,714 particles were selected. After rounds of 3D classification (C1 and C6 symmetry), a class with helical features was selected. The final 9.3 Å map of the TM region was reconstructed with ctf- and aberration-refined 6499 particles, followed by postprocessing (Supporting Information Figure S5). Subsequently, the TM region was resolved to 8.2 Å by applying a loose mask on the RHC FtsH-PD map (Supporting Information Figure S6).

### Structure Modeling of FtsH-PD and FtsH-TM

The FtsH-PD (PDB: 9wus) structure was modeled by molecular replacement using the MolRep in the spherically averaged phased translation function mode within the CCP-EM suite.^21^ The structural model of the periplasmic domain of FtsH in the FtsH–HflKC structure (PDB: 7wi3) was used as a template.^22^ One FtsH-PD subunit in the 7wi3 structure was used as an input, and six copies were modeled by molecular replacement. Then, it was refined by the real space refinement tool in Phenix^23^ and Refmac in the CPPEM suite.^24^ Manual buildings and adjustments were carried out in Coot.^25^ The final FtsH-PD model was validated using Phenix validation tools (Supporting Information Table S2). As the maps of the FtsH-TM region were not sufficient for structural model building, the hexameric-predicted model of FtsH, generated by AlphaFold2,^26^ run through ColabFold,^27^ was fitted into FtsH-TM maps using ChimeraX.^28^ Periplasmic and transmembrane regions of the predicted model were used only for fitting.

## Results

### Periplasmic Domain of FtsH as a Degradation Product of FtsH–HflKC Overexpression

The periplasmic domain of FtsH (FtsH-PD) was purified and characterized after the overexpression of FtsH–HflKC in *E. coli*. Cell membranes were first solubilized in DDM, and FtsH–HflKC was purified by pulling down the streptactin tag on FtsH. The purified complex was then transferred to Amphipol A8-35 and further purified using size exclusion chromatography (Supporting Information Figure S2A). SDS-PAGE analyses of the affinity-purified and Amphipol reconstituted complex both suggested that FtsH undergoes a degradation during the purification (Supporting Information Figure S2F). A strong band corresponding to the cytosolic domain of FtsH (∼60 kDa) was persistent during all stages (Supporting Information Figure S2F). Additionally, the band corresponding to HflC disappeared during the SEC step (Supporting Information Figure 2F, shown with an arrow). Consequently, the fraction used for cryo-EM data collection was enriched with the Amphipol-reconstituted periplasmic domain of FtsH, due to FtsH degradation and loss of the FtsH-CD and FtsH–HflKC dissociation. The negative-stain EM grid of this fraction showed uniform circular particles, which are ∼10 nm in diameter (Supporting Information Figure S2D,E).

### FtsH Periplasmic Domain Reveals Two Conformations

Cryo-EM analysis of the sample resulted in hexameric star-shaped 2D classes with a large Amphipol belt. 2D class averages of FtsH-PD initially suggested three different orientations and conformations. The first is the one with alpha helices oriented at the clockwise (CW) rotation, the second is the one with alpha helices oriented at the counterclockwise (CCW) rotation, and the third is the one with rotated-helix conformation (RHC) (Supporting Information Figure S1). After several particle subtraction and global and focused 3D classification steps, we finally obtained three different maps representing three different orientations. Upon careful consideration, we realized that the map with the CW rotation is a mirror image of the CCW rotated map. Therefore, the CCW map comprised right-handed helices, whereas the CW revealed left-handed helices, which is biologically unfavorable, however possible computationally as an artifact of absolute hand estimation during 3D reconstruction. The CW map flipped along the *z*-axis aligned well with the CCW map, allowing us to merge these particles to refine a single right-handed consensus structure.

The 4.9 Å CCW FtsH-PD cryo-EM structure (FtsH-PD-CCW) showed the same α + β fold as the NMR^29^ (pdb 2muy) and X-ray crystal structure^29^ (pdb 4v0b), as well as the periplasmic domain of FtsH obtained within the FtsH–HflKC cryo-EM structures^22,30^ (pdb 7vhp, 7wi3) comprising two α-helices and five β-strands (Figure 1D, Supporting Information Figure S7).^31^ In the hexameric structure, α-helix 1 (α1) and α-helix 2 (α2) have orientations that exhibit counterclockwise rotation (Figure 1C, purple map).

The third type of map had neither a CW nor a CCW orientation, which we named “rotated-helix conformation (RHC)”. The resolution of this map was 7.3 Å. We could not generate a reliable structural model for this EM map (Figure 1C, turquoise map). It is clear, however, that the α1 and α2 helices undergo a conformational change, adopting a novel conformation. Within the FtsH-PD, the α1 and α2 helices undergo a 20° clockwise rotation compared to the FtsH-PD-CCW structure (Figure 1C, superposed maps, Supporting Information Animation).

### FtsH Transmembrane Domain

The cryo-EM map of the FtsH transmembrane domain (FtsH-TM) was reconstructed using the particles subtracted from the 2D classes corresponding to the transmembrane region. The 9.3 Å map of the computationally isolated FtsH-TM (EMD-66013) revealed hexameric alpha helices. The TM domain is 48 Å in length, 45 Å in diameter (Figure 1E, gray map). The FtsH-TM was also resolved by using a loose mask on the FtsH-RHC map, which reached up to 8.2 Å (Figure 1E, salmon map). Two alpha helices in the TM domain were seen in this map. The hexameric AlphaFold2 model of the FtsH-TM and FtsH-PD was fitted in both maps.

### ATP Binding May Cause Conformational Changes in the Periplasmic FtsH

While FtsH is known to adopt different conformations upon ATP binding, current structural knowledge is largely restricted to the cytosolic domain (FtsH-CD). Because no high-resolution full-length structure exists, the influence of cytosolic conformational changes on the periplasmic domain remains structurally unverified. Our hypothesis that the observed FtsH-PD states may be coupled to the nucleotide cycle is supported by the addition of 0.2 mM ATP in the growth medium, which improved protein yields and potentially stabilized distinct states. However, because FtsH-CD was lost during sample purification and was not detected in our cryo-EM maps, we cannot structurally confirm the nucleotide-bound state of these particles. Consequently, the observed 20° rotation is presented here as an intrinsic capability of the periplasmic domain that might be functionally linked to the broader ATPase cycle.

### Possible Scenarios on FtsH Degradation Mechanism

Hypotheses about how FtsH degrades substrate proteins have been reported in the literature^7,10,11,22,32–35^ (Figure 2). The best-known hypothesis is that both soluble and membrane protein substrates enter the degradation chamber formed by hexamers through a pore between the cytosolic and transmembrane regions. Cytosolic protein substrates may be recognized by the helical subdomain of the ATPase domains, whereas membrane-bound substrates may interact with the FtsH transmembrane region through their transmembrane parts. Substrate recognition is followed by the translocation of both types of substrates into the protease domain for endoproteolysis. Recent studies proposed new supporting hypotheses: According to the study by Qiao et al., HflKC may provide an alternative entry route for periplasmic substrates via the pore region located on the top of the FtsH–HflKC complex on the periplasmic side.^22^ Ghanbarpour et al. suggest that FtsH in the FtsH–HflKC complex has lipid–scramblase activity which is also known as lipid flip-flop.^35^ This activity facilitates the movement or rearrangement of the membrane lipids across the bilayer. Therefore, the membrane integrity is maintained through this process and it also facilitates cell growth. It has been reported that this activity also enhances the capacity of FtsH to extract and degrade membrane-bound substrates. Our cryo-EM maps, revealing conformational changes in the periplasmic FtsH structure, provide a structural framework that is compatible with these two recent hypotheses. We hypothesize that the FtsH periplasmic domain may undergo conformational changes to aid the translocation of periplasmic substrates through the central pore of the periplasmic region of FtsH to reach FtsH’s cytoplasmic ATPase domain. The curved lipid domains in the FtsH–HflKC structures may be indeed related to the degradation of membrane-embedded substrates; where substrate binding and translocation may induce conformational changes in the transmembrane and periplasmic domains of FtsH.

**Figure 2:**
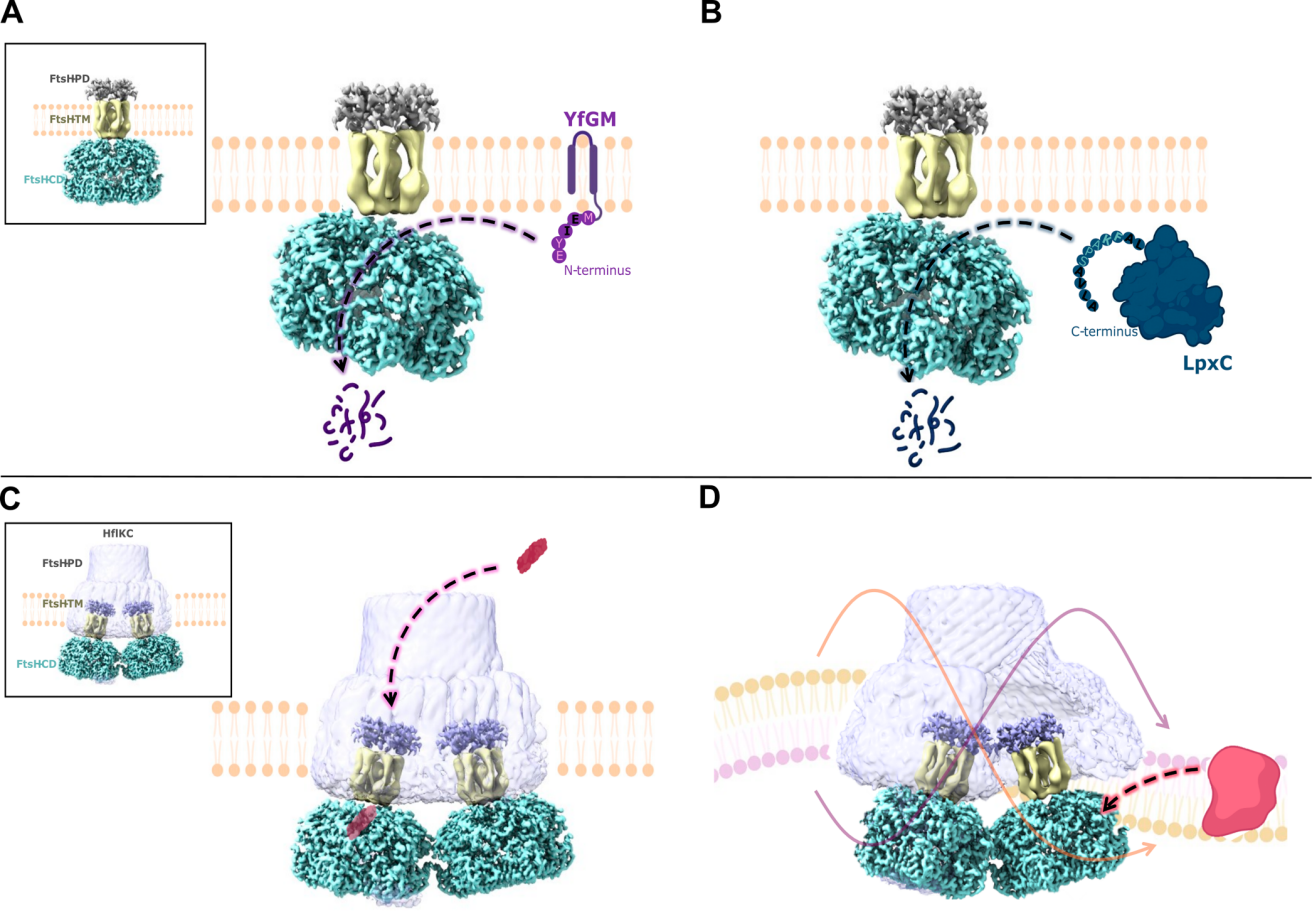
Proposed mechanisms for FtsH-mediated substrate protein degradation. (A) Cytoplasmic interaction of membrane substrates with FtsH. Recognition and unfolding of membrane substrates, such as YfgM, occur in the cytoplasm. (B) Cytoplasmic interaction of cytosolic substrates with FtsH. Cytosolic substrates, such as LpxC, are similarly recognized and unfolded in the cytoplasm prior to degradation. (C) Interaction of periplasmic substrates with FtsH. Periplasmic substrates (indicated in red) are proposed to access FtsH through a gap in the HflKC complex within the periplasmic space, followed by degradation. (D) Lipid scrambling activity by FtsH-HflKC. Lipid scrambling activity of FtsH–HflKC (purple and orange lipids and arrows) is suggested to facilitate substrate degradation of transmembrane substrates (red). FtsH PD and TM: this study, FtsH CD: EMD-32521,^22^ HflKC: EMD-46057.^35^

## Discussion

Widely conserved across bacteria and eukaryotic organelles, FtsH is a versatile protease with a large spectrum of substrates. It is involved in many cellular processes, particularly protein quality control mechanisms that are essential for cell viability. As a membrane-bound protease, FtsH can degrade both cytosolic and membrane proteins. While recent studies have provided static structural insights into FtsH, structural information detailing its conformational dynamics remains limited. Crystal structures of bacterial FtsH^10–12,36^ and recent cryo-EM structures^22,30^ were reported; however, these studies primarily captured single structural states, and a comprehensive understanding of the full-length FtsH conformational cycle is still lacking. While crystallization of the whole FtsH is a known challenge, obtaining a good cryo-EM sample of either FtsH or FtsH–HflKC complex is equally challenging.^7,22,30^ The dynamic nature of FtsH and its autocleavage activity need to be overcome to achieve a full-size, atomic resolution FtsH structure. The structures reported here provide the first evidence of conformational heterogeneity within the isolated periplasmic domain of FtsH, distinguishing our findings from previously reported single-state models. To the best of our knowledge, it is the smallest membrane protein reconstruction reported to date using a 200 kV cryo-electron microscope, along with the 3.68 Å structure of multidrug efflux pump in *Mycobacterium tuberculosis* ^37^ (49 kDa) and 3.7 Å structure of glutamate transporter homologue^38^ (44 kDa).

FtsH research focused on the cytosolic regions in which the proteolytic and ATPase domains of the protein are located. The periplasmic region of FtsH may be involved in substrate recognition, translocation, and degradation of proteins in the periplasm. FtsH is capable of degrading membrane-embedded substrates in either the N-to-C or C-to-N direction.^32,39^ It was shown, however, that the proteolysis of the periplasmic domain of the model substrate PhoA by FtsH was halted after degradation of the C-terminal cytosolic region.^32^ This raises the possibility that the degradation of periplasmic substrates is actually initiated and mediated by the FtsH–HflKC complex through the gap formed by the HflKC periplasmic domain. Substrate binding is suggested to be followed by translocation of substrates through the periplasmic region of FtsH, reaching the cytosolic proteolytic domain.^40^ Although HflKC has long been accepted as a regulator of FtsH proteolytic activity,^41,42^ FtsH–HflKC was shown to also degrade proteins,^22,30^ aligning with our experimental results (Supporting Information Figure S7). A recent study has proposed that the membrane curvature in the FtsH–HflKC complex is related to lipid–scramblase activity. Lipid scrambling would promote the degradation of membrane-embedded proteins by furthering interactions between membrane-bound substrates and the transmembrane region of FtsH.^35^ Based on our structural findings, we hypothesize that membrane protein substrates could thus reach the proteolytic active site of the FtsH via the gap formed by the hexameric periplasmic and transmembrane regions, a process that might be facilitated by conformational changes in these regions. Conformational change in the FtsH periplasmic region from CCW to RHC (Figure 1C) provides a structural basis to propose that such dynamics may be related to membrane-bound substrate movement in the membrane and degradation, although this requires future functional validation. While in vivo biochemical studies are strictly needed to confirm this, it is tempting to hypothesize that these structural changes, along with the reported scramblase activity in FtsH–HflKC complexes, could potentially aid in substrate extraction by reducing the energy barrier for substrate dislocation. This mechanism is consistent with research suggesting that the degradation of periplasmic substrates such as YfgM requires cytosolic N-terminal recognition,^43^ implying a coordinated ’pull and translocate’ process in which initial substrate interaction in the cytosol is followed by unfolding. Notably, the inability of FtsH to degrade the periplasmic domain of PhoA, unless initiated from the cytosolic end,^32^ supports a molecular mechanism in which HflKC forms a protected periplasmic substrate channel in its vault-like architecture (Figure 2C).

The retention of proteolytic activity in the FtsH–HflKC complex, as demonstrated in this study and by Qiao et al. (2022), contradicts previous reports suggesting that HflKC is a down-regulator of FtsH proteolytic activity.^3,22,44^ Thus, HflKC may act as a topological gatekeeper, limiting substrate access to FtsH and preventing indiscriminate degradation while allowing processive threading of membrane-embedded substrates. This proposed regulatory mechanism is consistent with our observation of dynamic conformational changes in the periplasmic domain (Figure 1C), leading to the hypothesis that the RHC state might represent an intermediate step that may be cooperating with HflKC in directing substrates toward the FtsH cytoplasmic active site.

Recent research demonstrating lipid–scramblase activity in FtsH–HflKC suggests a mechanism linking substrate dislocation to membrane remodelling, with asymmetric lipid distribution aiding the extraction of transmembrane domain (TM)-anchored substrates.^35^

The dual role of ATP as a proteolytic activator and destabilizing factor adds complexity for such mechanistic models: For instance, while ATPγS stabilization was critical for structure determination, native ATP hydrolysis likely drives rapid conformational changes in FtsH (>6–8 ATP molecules per proteolytic event).^10^ Furthermore, the autocleavage ability of FtsH highlights the importance of HflKC in maintaining FtsH and FtsH–HflKC complex integrity during substrate processing, a regulatory mechanism observed in other AAA+ proteases.^43^

It is important to note that while our cryo-EM data capture the intrinsic conformational flexibility of the FtsH periplasmic domain, the mechanistic models discussed herein remain structurally informed hypotheses. The 7.3 Å resolution of the new state confidently establishes a secondary structure rearrangement but does not provide the atomic detail required for full mechanistic validation. Future functional genetics, mutagenesis, and in vitro biochemical assays will be essential to map how the observed RHC state couples to substrate unfolding and lipid scrambling. There are unanswered questions regarding the mechanisms of degradation of membrane-embedded or periplasmic substrates by FtsH alone or in complex with HflKC. How the periplasmic region of the FtsH is involved in this process requires further research. High-resolution structures of full-length FtsH, as well as substrate-bound FtsH and FtsH–HflKC complexes, along with biochemical and functional experiments will enhance our understanding of the molecular mechanisms of FtsH substrate recognition, translocation, and degradation. Although further high-resolution studies will be needed to clarify the exact molecular mechanisms underlying this new conformation, the 7.3 Å resolution map confidently establishes a secondary structure rearrangement. This newly observed conformation suggests a highly flexible conformational landscape that might be functionally relevant and improves the known structural “store” of FtsH.

## Supplementary Material





## Data Availability

All cryo-EM maps generated have been deposited to Electron Microscopy Data Bank (EMDB) as entries EMD-66269 (FtsH periplasmic domain), EMD-66013 (FtsH periplasmic domain in rotated-helix conformation (RHC), EMD-66015 (FtsH transmembrane domain), EMD-66014 (FtsH transmembrane and periplasmic domain), and in the Protein Data Bank (PDB) as PDB entry 9WUS (FtsH periplasmic domain).

## Notes

Cryo-EM workflow of FtsH; cryo-EM data collection parameters; FtsH-PD refinement process; purification and sample preparation of FtsH for EM; resolution of the FtsH-PD map according to FSC; resolution of the FtsH-PD-RHC map according to FSC; resolution of the transmembrane domain from the FtsH-PD map according to FSC; resolution of the transmembrane and periplasmic domains from the FtsH-PD-RHC map according to FSC; ATPase and protease activities of FtsH–HflK; and superposition of *E. coli*FtsH periplasmic domain structures PDF)
Supporting Information Animation (GIF)

## #

University Medical Center Hamburg-Eppendorf, Institute of Biochemistry and Signal Transduction, Hamburg, 20246, Germany
