# Correction to “Cryo-EM Structure of the FtsH Periplasmic Domain Reveals Functional Dynamics”

**DOI:** 10.1021/acschembio.6c00551

**Published:** 2026-06-25

**Authors:** Günce Göc, Sathish K. N. Yadav, George Orriss, Ufuk Borucu, Imre Berger, Christiane Schaffitzel, Burak V. Kabasakal

Corrections

1. Supporting Figures S5 and S6 appear to have been interchanged in the published Supporting Information PDF. The captions are consistent with the Methods section, but the figure panels themselves appear to be placed under the wrong figure numbers.

The correct assignment should be:

**Supporting Figure S5.** The resolution of the transmembrane domain from the FtsH-PD map according to FSC. **A.** Gold standard Fourier Shell Correlation (FSC) curves of the FtsH-TM maps. The 9.3 Å resolution was determined using the FSC = 0.143 criterion B. Local resolution map of FtsH-TM, varying between 8.5 and 9.7 Å.

**Supporting Figure S6.** The resolution of the transmembrane and periplasmic domains from FtsH-PD-RHC map according to FSC. **A.** Gold standard Fourier Shell Correlation (FSC) curves of the FtsH-PD-TM maps. The 8.2 Å resolution was determined using the FSC = 0.143 criterion **B.** Local resolution map of FtsH-PD-RHC and FtsH-TM, varying between 6.2 and 7.4 Å.

This assignment is also consistent with the Materials and Methods section of the main article.

2. There is an issue with Supporting Figures S7 and S8. In the Results section on page 847, the structural comparison of the FtsH-PD-CCW cryo-EM structure with previously published NMR, X-ray, and cryo-EM structures is cited as **Supporting Information Figure S7**.

The relevant structural superposition figure is currently labeled as **Supporting Figure S8** in the SI, whereas **Supporting Figure S7** currently contains the ATPase/protease activity data.

Since the structural comparison is cited before the functional assay data in the main text, we think the cleanest correction would be to reorder/renumber these two Supporting Figures as follows:

**Current Supporting Figure S8**, showing the structural superposition, should become **Supporting Figure S7.**

**Current Supporting Figure S7**, showing the ATPase/protease activity assays, should become **Supporting Figure S8.**

The later **Discussion** reference to the functional assay data (second paragraph in the Discussion section on Page 849) should then be updated from Supporting Information Figure S7 to Supporting Information Figure S8.

