# Supplementary material for: Cryo-EM Structure of the FtsH Periplasmic Domain Reveals Functional Dynamics
Source: ACS Chem Biol. 2026 Apr 7;21(4):844–51. doi: 10.1021/acschembio.5c01025 (PMC13097134; doi:10.1021/acschembio.5c01025)
Supplement: Supplementary file 1 [file cb5c01025_si_001.pdf]

Supporting Information

Cryo-EM structure of the FtsH periplasmic domain reveals functional dynamics

Günce Göc[1,2,‡], Sathish K. N. Yadav[3], George Orriss[3], Ufuk Borucu[3], Imre Berger[3,4], Christiane Schaffitzel[3] and Burak V. Kabasakal[1,3,5*]

[1] Turkish Accelerator and Radiation Laboratory, Ankara, 06830, Türkiye

[2] Kocaeli University, Department of Biology, Kocaeli, 41001, Türkiye

[3] School of Biochemistry, University of Bristol, Bristol, BS8 1TD, United Kingdom

[4] University of Bristol, School of Chemistry and Max Planck Bristol Centre for Minimal Biology, University of Bristol, Bristol, BS8 1TH, United Kingdom

[5] Department of Biological Sciences, Middle East Technical University, Ankara, 06800, Türkiye

[‡] Present address: University Medical Center Hamburg-Eppendorf, Hamburg, 20246, Germany

* To whom correspondence may be addressed

bvkabasakal@tarla-fel.org

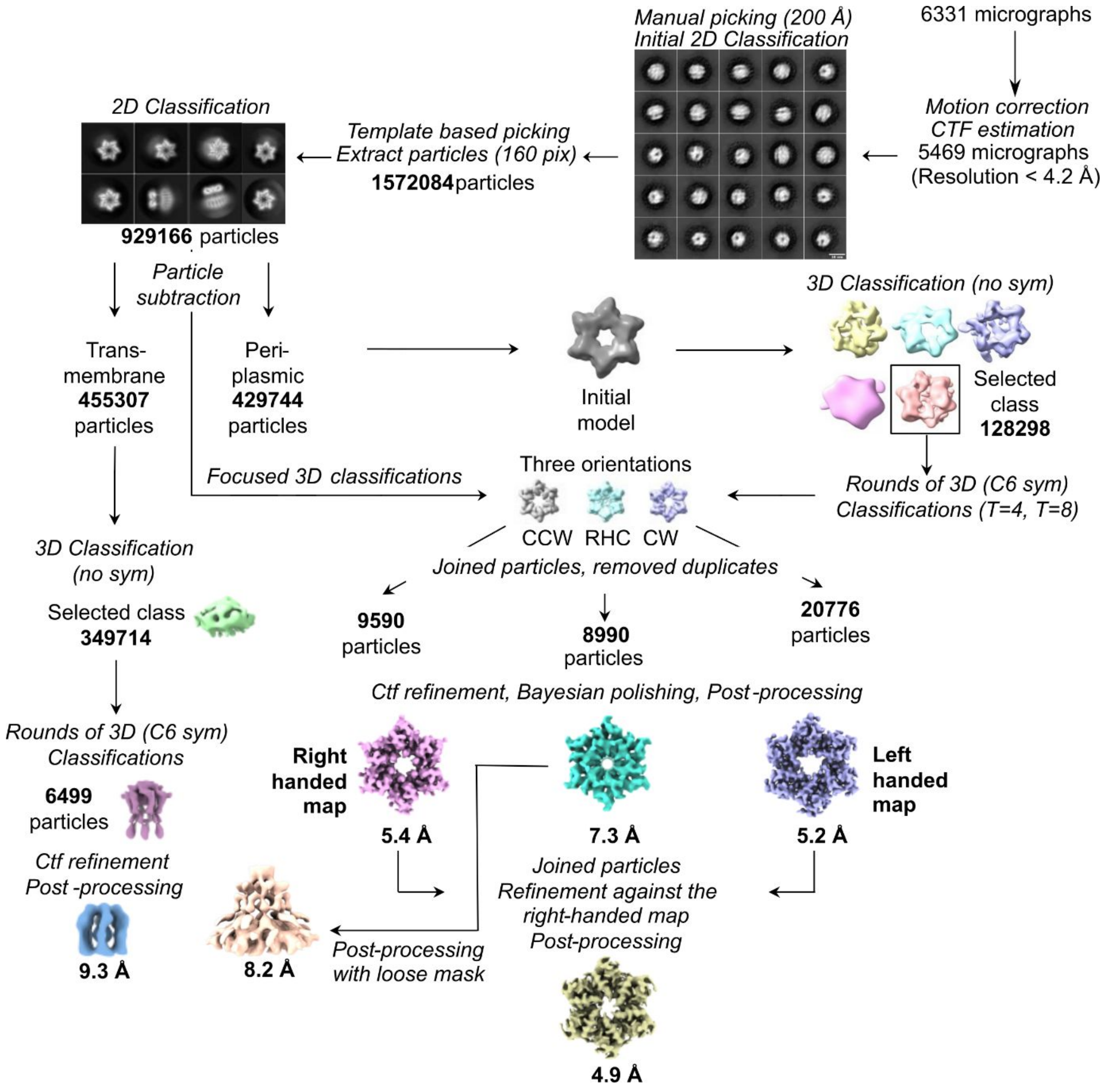

Supporting Figure S1. Cryo-EM workflow. FtsH periplasmic domain (PD) and transmembrane domain (TM) sub-regions were reconstructed separately by using subtracted particles extracted with masks of corresponding regions. The FtsH-PD region was reconstructed by focused 3D classification. Three different orientations were obtained for FtsH-PD; two of them (right-hand and left-hand) belong to a known FtsH-PD conformation, the third map exhibits a novel conformation (RHC: roated-helix conformation). The particles corresponding to left-handed (CW) and right-handed (CCW) maps were combined and refined to obtain the right-handed map. PD and TM regions were obtained in one map by applying a loose mask to the RHC map of FtsH-PD.

Supporting Table S1. Cryo-EM Data Collection

**Voltage (kV)**	200
**Nominal Magnification**	130,000
**Pixel Size [Å]**	1.05 (0.525)
**Total Dose [e^{-}/ Å^{2}]**	61.3

**Number of Fractions**	48
**Total Dose per Fraction [e^{-}/ Å^2]**	1.28
**Defocus Range [μm]**	-1 to -2
**No. of micrographs**	6331
**Initial particle images**	929,166
**Final particle images**	30,366
**Symmetry imposed**	C6

Supporting Table S2. FtsH-PD Refinement

**Initial mode used (PDB)**	7WI3
**PDB code**	9WUS
**EMDB code**	EMD-66269
**Composition**	
**Chains**	6
**Atoms**	3168
**Residues**	390
**Water**	0
**Ligands**	0
**Bonds (RMSD)**	
**Length (Å)**	0.002
**Angles (°)**	0.567
**B-factor (Å^2)**	125.9
**MolProbity score**	2.09
**Clash score**	12.5
**Ramachandran plot (%)**	
**Outliers**	0
**Allowed**	7.9
**Favored**	93.65
**Rotamer outliers (%)**	0
**CaBLAM outliers (%)**	4.9
**Resolution FSC (0.143)(Å)**	4.24
**CC (mask)**	0.65

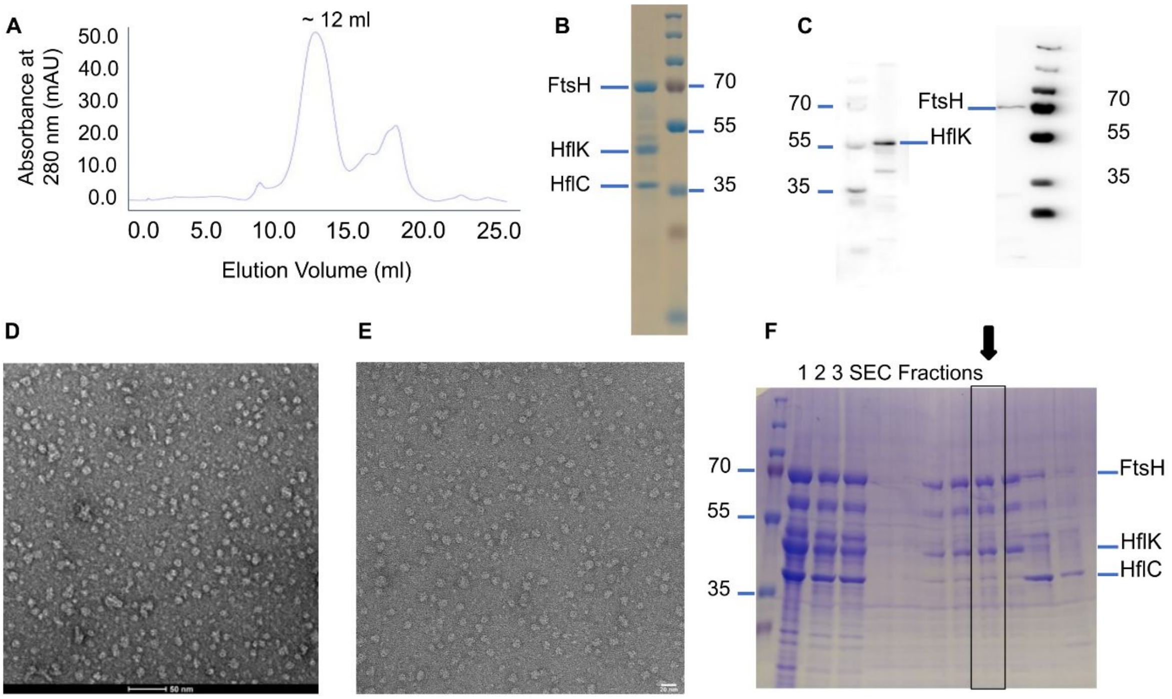

Supporting Figure S2. Protein purification and sample preparation for EM. A. Size-exclusion chromatography (SEC) of purified FtsH-HflKC complex. **B.** SDS-PAGE analysis of the peak fraction. **C.** Western Blot analyses of streptactin affinity-purified FtsH-HflKC. Anti-His and anti-Strep tag antibodies were used. **D.** NS-EM micrograph (Scale bar is 50 nm). **E.** Cryo-EM micrograph (Scale bar is 20 nm). **F.** SDS-PAGE analysis of Amphipol-reconstituted FtsH-HflKC. 1. After Streptactin affinity purification, 2. After complex reconstitution in Amphipol, 3. After detergent removal with Biobeads, SEC Fractions: Peak fractions during SEC. The highlighted fraction (box) was used for NS-EM and cryo-EM analysis.

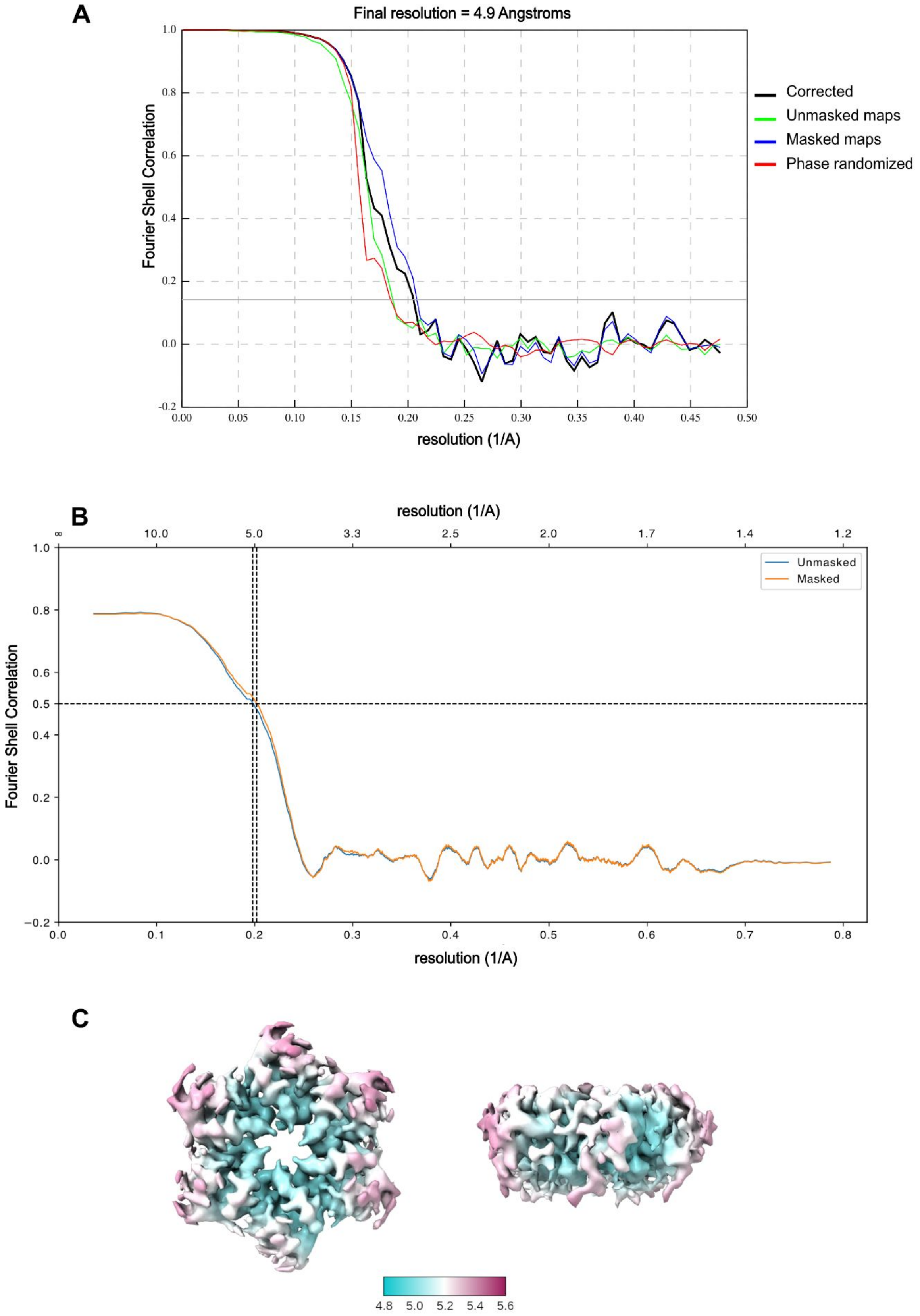

Supporting Figure S3. The resolution of the FtsH-PD map according to FSC. A. Gold standard Fourier Shell Correlation (FSC) curves of the FtsH-PD maps. The 4.9 Å resolution was determined using the FSC=0.143 criterion (shown in gray line). **B.** The model map FtsH-

PD FSC curve calculated between the model and the map. The resolution indicates 5.0 Å at FSC=0.5. **C.** Local resolution map of FtsH-PD, varying between 4.8-5.6 Å.

A

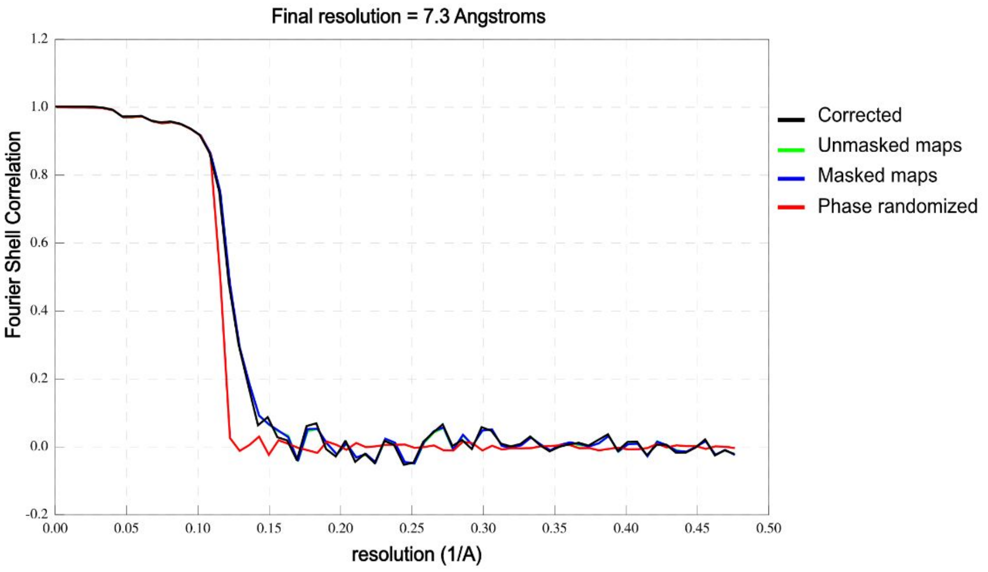

B

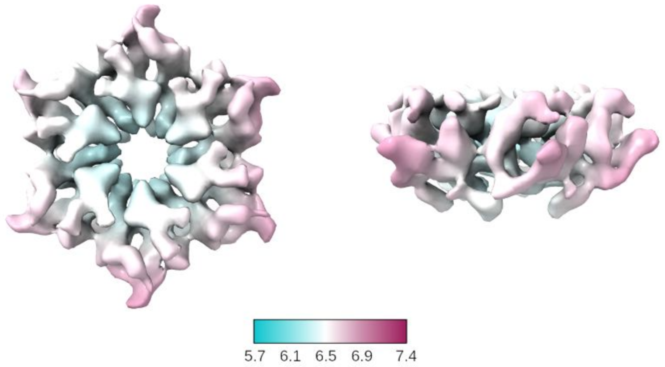

Supporting Figure S4. The resolution of the FtsH-PD-RHC map according to FSC. A. Gold standard Fourier Shell Correlation (FSC) curves of the FtsH-PD maps. The 7.3 Å resolution was determined using the FSC=0.143 criterion **B.** Local resolution map of FtsH-PD-RHC, varying between 5.7-7.4 Å.

A

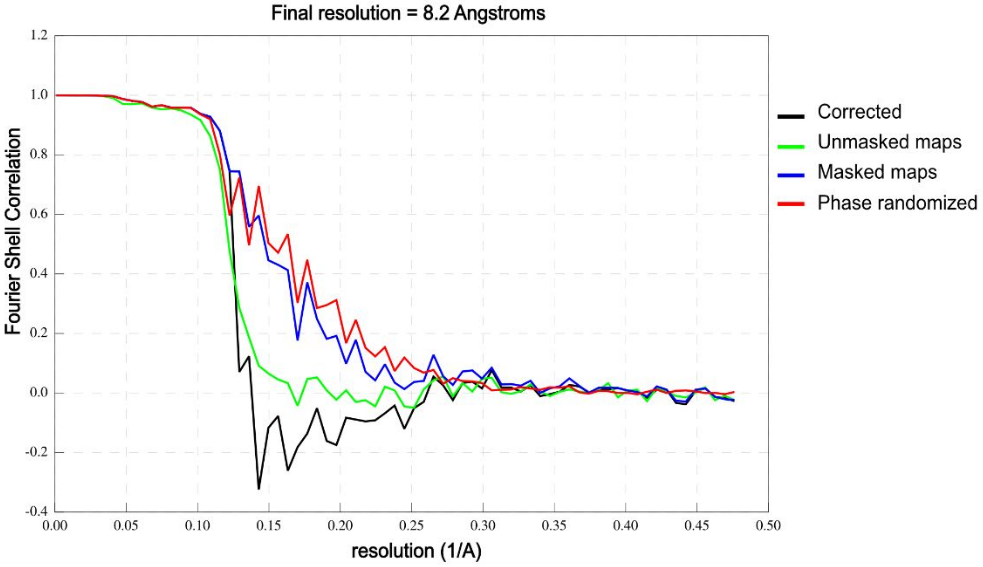

B

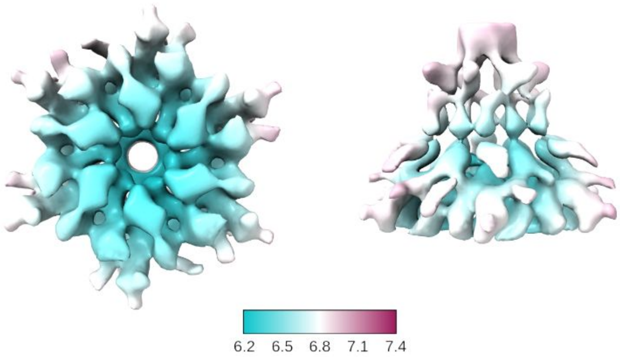

Supporting Figure S5. The resolution of the transmembrane domain from the FtsH-PD map according to FSC. A. Gold standard Fourier Shell Correlation (FSC) curves of the FtsH-PD maps. The 9.3 Å resolution was determined using the FSC=0.143 criterion **B.** Local resolution map of FtsH-PD-RHC, varying between 8.5-9.7 Å.

A

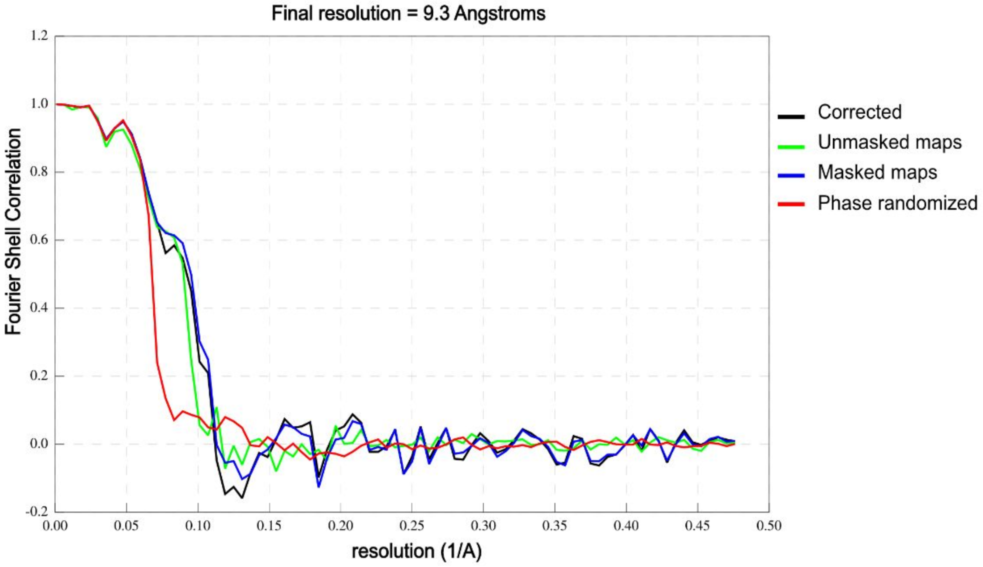

B

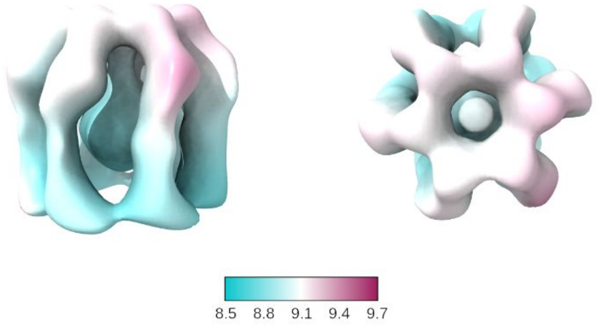

Supporting Figure S6. The resolution of the transmembrane and periplasmic domains from FtsH-PD-RHC map according to FSC. A. Gold standard Fourier Shell Correlation (FSC) curves of the FtsH-PD maps. The 8.2 Å resolution was determined using the FSC=0.143 criterion **B.** Local resolution map of FtsH-PD-RHC, varying between 6.2-7.4 Å.

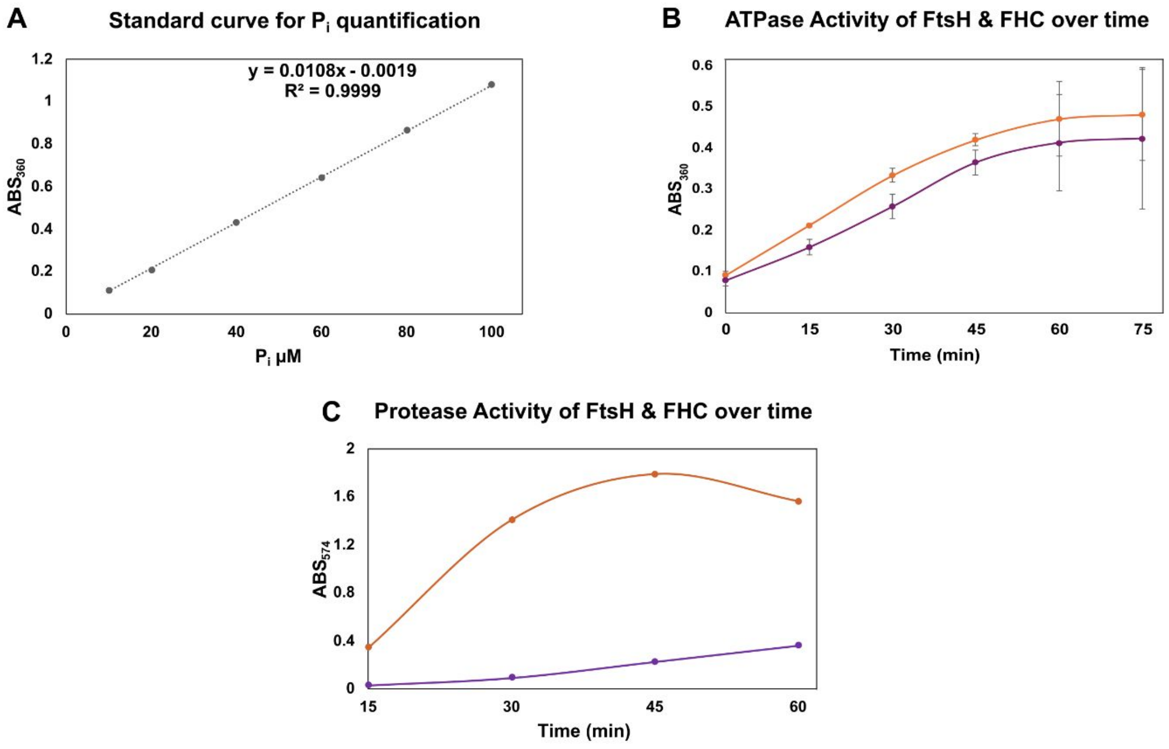

Supporting Figure S7. ATPase and protease activities of FtsH-HflKC. A. ATP hydrolysis was measured by quantifying inorganic phosphate (P_i) release over time, **B.** FtsH (orange) and FtsH-HflKC (FHC, purple) were incubated with 5 mM ATP, and P_i release was monitored, then subtracted from standard curve of P_i at the indicated time points. **C.** Protease activity of FtsH (orange) and FHC (purple) proteins using casein (0.4%) as substrate.

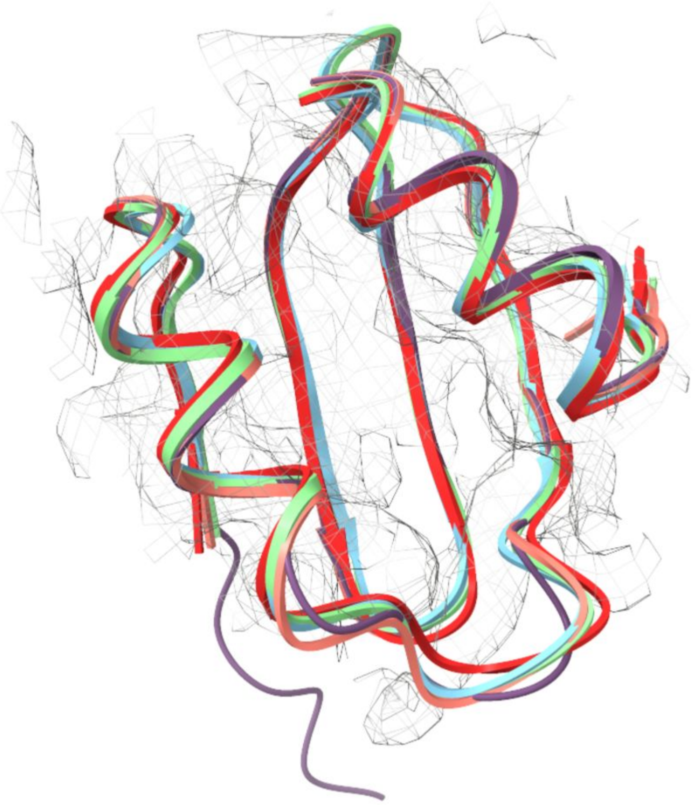

Supporting Figure S8. Superposition of *E. coli* FtsH periplasmic domain structures. NMR (pdb 2muY, red), X-ray (pdb 4V0B, turquoise), and cryo-EM structures (pdb 7wi3 and 7vhp, salmon and green, respectively) of FtsH periplasmic domains are aligned with the FtsH-PD structure (purple). FtsH-PD EM density is shown in grey mesh. All structures reveal the conserved α+β fold.
