# Supplementary figures and images for: Cryo-EM Structure of the FtsH Periplasmic Domain Reveals Functional Dynamics
Source: ACS Chem Biol. 2026 Apr 7;21(4):844–51. doi: 10.1021/acschembio.5c01025 (PMC13097134; doi:10.1021/acschembio.5c01025)

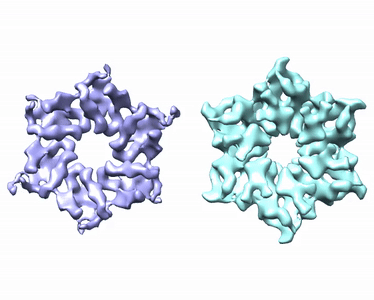

Supplement: Supplementary file 2 [file cb5c01025_si_002.gif]
